# “I Give It Everything for an Hour Then I Sleep for Four.” The Experience of Post-stroke Fatigue During Outpatient Rehabilitation Including the Perspectives of Carers: A Qualitative Study

**DOI:** 10.3389/fneur.2022.900198

**Published:** 2022-06-02

**Authors:** Erin D. Bicknell, Catherine M. Said, Kimberley J. Haines, Suzanne Kuys

**Affiliations:** ^1^School of Allied Health, Australian Catholic University, Brisbane, QLD, Australia; ^2^Department of Physiotherapy, Western Health, St Albans, VIC, Australia; ^3^Department of Physiotherapy, The University of Melbourne, Melbourne, VIC, Australia; ^4^Australian Institute for Musculoskeletal Science, St Albans, VIC, Australia

**Keywords:** stroke, fatigue, rehabilitation, qualitative, carers

## Abstract

**Background:**

Fatigue is a debilitating post-stroke symptom negatively impacting rehabilitation. Lack of acknowledgment from carers can be additionally distressing. The purpose of this study was to describe the experience of post-stroke fatigue during outpatient rehabilitation, including the perspectives of carers.

**Methods:**

This qualitative study was guided by descriptive phenomenology within a constructivist paradigm. Semi-structured interviews were conducted with stroke survivors experiencing fatigue (Fatigue Assessment Scale >23) and attending outpatient rehabilitation. Carers were also interviewed where identified, providing insight into their own and stroke survivor experiences. Data were analyzed according to Colaizzi's analytic method.

**Results:**

Fourteen stroke survivors (50% culturally and linguistically diverse), and nine carers participated. Six themes were identified: 1. The unpredictable and unprepared uncovering of fatigue; 2. Experience and adjustment are personal 3. Being responsible for self-managing fatigue; 4. The complex juggle of outpatient stroke rehabilitation with fatigue; 5. Learning about fatigue is a self-directed problem-solving experience; 6. Family and carers can support or constrain managing fatigue.

**Conclusion:**

Despite engaging in outpatient rehabilitation, stroke survivors largely learnt to manage fatigue independent of healthcare professionals. Carers often facilitated learning, monitoring rehabilitation, daily routines and fatigue exacerbation. Conversely, family could be dismissive of fatigue and possess unrealistic expectations. Post-stroke fatigue must be considered by clinicians when delivering outpatient rehabilitation to stroke survivors. Clinicians should consistently screen for fatigue, provide flexible session scheduling, and educate about individual indicators and strategies for management. Clinicians should also explicitly engage carers who play a critical role in the management of fatigue.

## Introduction

Post-stroke fatigue has been described as a feeling of “…tiredness, a lack of energy, or an increased need to rest… (which) has led to difficulty taking part in everyday activities.” (1) (p. 543). Fatigue is associated with dependence in daily activities, poor quality of life (2), reduced physical activity (3), and increased morbidity and mortality (4). Approximately half of all stroke survivors experience fatigue (5), regardless of severity (6). Optimizing motor recovery post-stroke requires high-dose rehabilitation (7, 8). However, stroke survivors report fatigue impacts recovery (9), limiting therapy participation and independent practice (10, 11). Little is known about how people participating in outpatient rehabilitation experience fatigue. This is a challenging time when stroke survivors are adjusting to new disabilities, adapting to life at home while continuing with intensive rehabilitation. Clinicians are not providing sufficient education and support for post-stroke fatigue (12, 13). Understanding how fatigue impacts stroke survivors and their carers while participating in outpatient rehabilitation will assist health professionals to support stroke survivors and equip them and their carers with knowledge and skills to manage.

A systematic review of qualitative studies conceptualized the experience of fatigue in stroke survivors into five core characteristics; lack of energy to perform activities, abnormal need for sleep, becoming easily tired, feeling fatigue is unpredictable, and increased stress sensitivity (12). Interestingly, no themes emerged around fatigue and rehabilitation, as most studies recruited participants in the chronic phase of stroke recovery. One study explored stroke survivor experience of fatigue specifically during inpatient rehabilitation. While factors particular to the hospital environment, such as ward regime and noise were identified as exacerbating fatigue, the relationship between rehabilitation and fatigue was not explored and did not emerge (14). It is possible this experience differs when stroke survivors return to their daily environment whilst also engaging in rehabilitation.

Additionally, stroke survivors appear to cope better if their fatigue is acknowledged and supported and feel increased emotional distress when carers lack appreciation of fatigue (12). Further investigation of carer perspectives is required to understand factors that influence fatigue acknowledgment and carer burden of post-stroke fatigue, with carers identifying this as a research priority (13, 15).

This study aims to describe the experience of post-stroke fatigue during outpatient rehabilitation in a group of stroke survivors and their carers.

## Methods

### Design

This qualitative study employed a descriptive phenomenological approach, within a constructivist paradigm. Phenomenology is concerned with obtaining the lived-experience—the essence, or true meaning of a phenomenon (16). Descriptive phenomenology supported the physiotherapist researcher to describe the experience in an unbiased way (17). Bracketing and separating preconceived beliefs out of consciousness were used to capture the essence of the experience in its purest form (18). In the constructivist paradigm, description of the experience is accessed through the construction of knowledge obtained from the view of the person perceiving the experience (19). Ethics approval was obtained from the participating facility (HREC/18/WH/47137) and university (2019-17R) ethics committees. This study is reported using the Standards for Reporting Qualitative Research (20). Throughout, *healthcare professional* refers to professionals involved in stroke survivor health care, and *rehabilitation service* refers to facilities providing rehabilitative care.

### Participants

Stroke survivors who experienced fatigue were purposively sampled inclusive of a range of ages, stroke severities and cultural backgrounds from a cohort attending physiotherapy at an outpatient rehabilitation facility that has two sites in Melbourne, Australia. Residents in the geographical area are from culturally and linguistically diverse backgrounds—~25% of stroke survivors admitted do not speak English as their primary language (21).

Stroke survivors were screened and invited to participate by their treating physiotherapist if they met the following inclusion criteria:

- 18–75 years of age.
- Participated in a minimum five hours face-to-face therapy per week for greater than two weeks (primary outpatient rehabilitation period).
- Scored >23 on the Fatigue Assessment Scale (22).
- Could provide informed consent.
- Able to articulate their experience of fatigue independently or with support from their carer.

Stroke survivors with other health conditions causing fatigue, such as cancer or chronic respiratory conditions were excluded. The Fatigue Assessment Scale was completed by treating physiotherapists when screening their existing caseload and new admissions for the study. Upon meeting inclusion criteria, the stroke survivor was immediately referred to the study. Stroke survivors were asked if they identified as having a carer and were provided the option of their carer attending the interviews. There were no other carer inclusion or exclusion criteria.

### Data Collection

**Figure 1:**
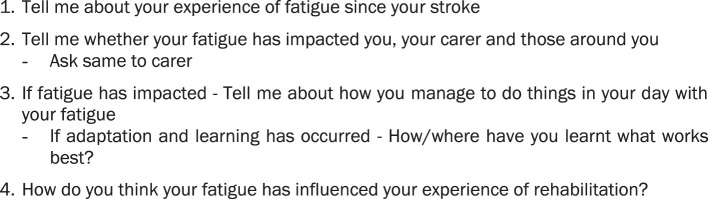
Interview questions.

Face-to-face semi-structured interviews were conducted by the primary researcher (EB) and audio recorded. An open-ended, pre-piloted interview schedule was used to elicit perceptions of stroke survivors, together with their carers where present (Figure 1). Pilot testing of the interview schedule, conducted with a stroke survivor and carer not included in the study, resulted in minor changes. No changes were made to the interview schedule between participants. Participants could share what they wanted in the interviews, with probes seeking further depth and clarification (23) and minimizing the researcher imparting assumptions (24). Carers provided further insight into stroke survivors' experiences as well as their own.

Researcher thoughts and participants' non-verbal communication were documented. Interviews of 30–60 min were conducted in a private room at the facility or participant's homes, between March-December 2019. Professional interpreters were utilized at the preference of the participant. Potential participants with stroke-related communication impairment were seen by a Speech Pathologist as part of routine care, to determine suitability for interview. Interviews were conducted by a clinician with substantial experience communicating with people with aphasia. Verbal communication in the interview was adapted to be aphasia friendly (25). This included modifying questions as required, being patient and encouraging, noting non-verbal communication, allowing participants to access the questions and write responses before the interview, and having carers present to support communication. Interview questions were provided to participants one week prior to their interview (26).

### Data Analysis

**Figure 2:**
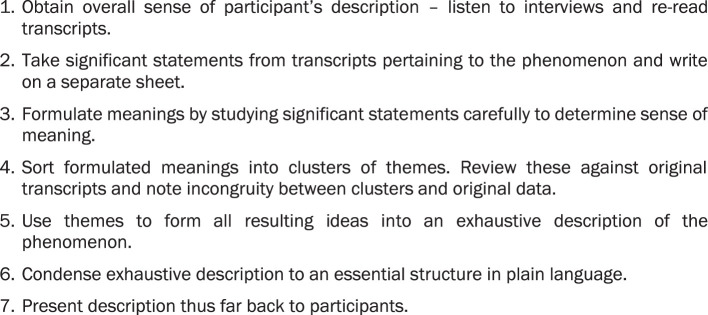
Colaizzi's method (28).

Audio recordings were transcribed verbatim by the primary researcher (EB) to increase data familiarity. Anonymised transcripts were exported to the encrypted web-based qualitative data-analysis application Dedoose (27). Comments recorded in the interview schedule were added to the transcripts. Data were analyzed after blocks of four to six interviews according to Colaizzi's seven-step, non-linear analytic method (28) (Figure 2), aligned with descriptive phenomenology (29). To enhance credibility of results (30), the essential structure was presented to participants and carers at a second interview either face-to-face or over the phone. Participants confirmed if their experience was accurately depicted and provided elaboration of data, but no new themes emerged. Recruitment continued until study size was sufficient to elucidate the study's aims (31), applying the concept of information power whereby study aim, specificity of information participants hold, theoretical background, quality of dialogue, and data analysis strategy are continually considered in guiding sample size (32). Prior research about the topic, including a theoretical model for post-stroke fatigue (33) informed development of the study aim and research questions. Quality of dialogue was strengthened due to the primary researcher's extensive experience communicating with stroke survivors, including those with aphasia (32). Sample size was expected between six and twenty, as is commonly necessary for adequate information power in phenomenological studies (17). No new knowledge emerged relevant to the study's aims in the final three interviews.

All researchers contributed to study design. Colaizzi's method was executed by a novice in qualitative research (EB) who underwent tertiary provided training, interview observation in another study, and received practical mentorship from the research team. Data analysis was independently confirmed by another researcher with extensive experience in qualitative research (SK). The decision trail at each step of the research process was audited and emerging results and information power challenged by two other members of the research team (CS, KH) with experience in rehabilitation (CS in stroke specifically) and in qualitative research (29). Final themes and illustrative quotations were critically analyzed for verification (CS, KH). The researcher conducting data collection (EB) had 10 years' experience in stroke rehabilitation. Participants were not patients of the researcher but may have had indirect contact during their rehabilitation. Continual reflexive analysis of the researcher's impact on the research process was addressed through journaling and discussions with the research team (SK, CS), to assist with debriefing and bracketing.

## Results

Fifteen stroke survivors were approached; one declined to participate. Four female and ten male stroke suvivors (43–71 years; ten infarct, four hemorrhage), and six female and three male carers were recruited. Carers, where identified, were all the significant other living with the stroke survivor. Seven participants were born outside Australia. Languages spoken other than English were Egyptian Arabic, Filipino, Macedondian, Turkish and Chin Hakha. Formal interpreters were utilized for the latter three languages whilst the other participants elected not to access an interpreter. Further details regarding participant demographics can be found in Table 1.

**Table 1:** Demographic and health information.

Participant*	**Sex**	**Age**	**Stroke type**	**Time post stroke (months)**	**Work status**	**Aphasia^**∧**^**	**Identified carer? Participated in interview?**	**Mobility**	**FAS**
John	M	64	Infarct R MCA with ECR	10	Retired	No (dysarthria)	Yes and at interview	Independent nil aid 1 km	46/50
Ivan	M	47	Infarct L PICA	3	Part-time (graded RTW)	No	No	Independent nil aid 1 km	26/50
Rose	F	70	Infarct L MCA with ECR	4.5	Homemaker	Mild	Yes and at interview	Independent nil aid 500 m	29/50
Lachlan	M	61	Hemorrhage R frontal & parietal lobe	7	Extended leave	No	No	Independent with walking stick 500 m	29/50
Bernard	M	67	Infarct R MCA with ECR, SAH + ICH	14	Retired	Mild	Yes and at interview	Supervision with walking frame 200 m + foot up	25/50
Kapil	M	46	Hemorrhage R cerebellum parenchymal	5	Extended leave	Moderate	No	Independent nil aid 1 km	29/50
Ahmed	M	43	R ACA infarct	10	Extended leave	Mild	Yes and at interview	Supervision 500 m	33/50
Neva	F	60	L basal ganglia ICH	8	Extended leave	Mild	Yes but not at interview	Supervision 500 m	24/50
Francesco	M	62	R cerebellar vermis hemorrhage	3	Extended leave	No	Yes and at interview	Close supervision 50 m	24/50
George	M	68	R MCA infarct	3	Retired	No	Yes and at interview	Independent nil aid 2 km	31/50
Olga	F	71	R MCA infarct	6.5	Retired	No	Yes and at interview	Independent with walking frame 100 m	31/50
Maida	F	70	L MCA infarct	3.5	Homemaker	Mild	Yes and at interview	Independent with walking stick 250 m	37/50
Danny	M	45	L paraventricular + frontal lobe infarcts	2	Extended leave	No	Yes but not at interview	Independent nil aid 1 km	34/50
Jericho	M	67	L frontal lobe infarct	4.5	Retired	Moderate	Yes and at interview	Low turn transfer, not walking	30/50

ACA, Anterior Cerebral Artery; ECR, Endovascular Clot Retrieval; FAS, Fatigue Assessment Scale; ICH, Intracerebral Hemorrhage; L, Left; MCA, Middle Cerebral Artery; PICA, Posterior Inferior Cerebellar Artery; R, Right; RTW, Return to work; SAH, Subarachnoid Hemorrhage; *Names are pseudonyms. ^∧^As deemd by Speech Pathologist.

**Figure 3:**
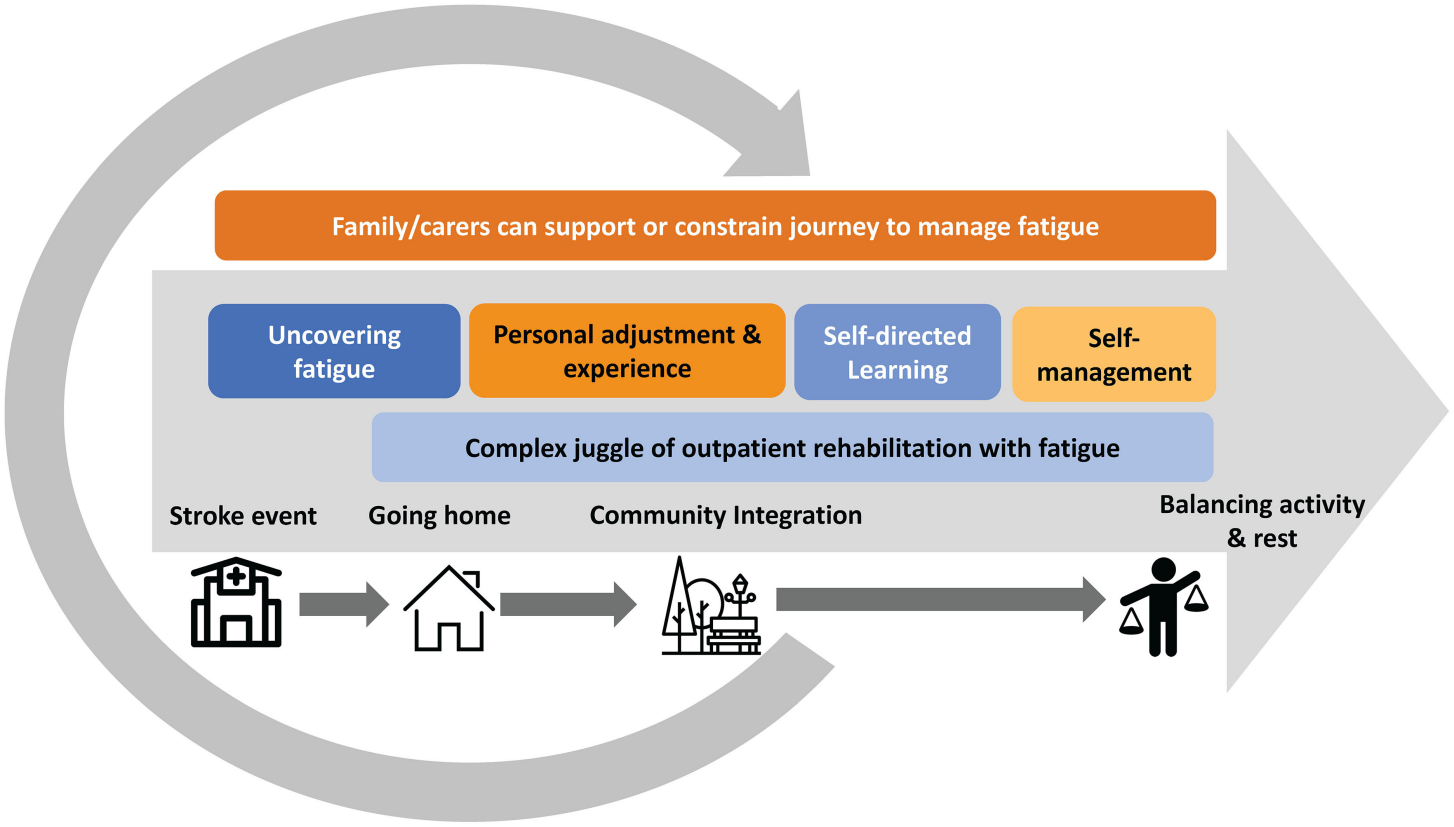
Figurative representation of the experience of post-stroke fatigue during outpatient rehabilitation. The six themes are presented in a timeline from stroke onset, time in hospital, discharge home and integration into the community. Themes 1, 2, 3 and 5 have chronological attributes. *Theme 1—The unpredictable and unprepared uncovering of fatigue* is first experienced when returning home, *Theme 2—Experience and adjustment are personal* represents the next phase where stroke survivors are grappling with adjustment prior to learning more about fatigue and engaging in self-management; *Theme 3—Being responsible for self-managing fatigue* and *Theme 5—Learning about fatigue is a self-directed problem-solving experience*. *Theme 4—The complex juggle of outpatient stroke rehabilitation with fatigue* coincides with the above, commencing once home and starting outpatient rehabilitation, and *Theme 6—Family and carers can support or constrain managing fatigue* is presented outside the timeline, and overlapping, as it had an influence on all other themes.

Six themes were identified describing the experience of post-stroke fatigue during outpatient rehabilitation (Figure 3): 1. The unpredictable and unprepared uncovering of fatigue; 2. Experience and adjustment are personal; 3. Being responsible for self-managing fatigue; 4. The complex juggle of stroke rehabilitation with fatigue; 5. Learning about fatigue is a self-directed problem-solving experience; 6. Family and carers can support or constrain managing fatigue. Excerpts illustrating themes are presented in Supplementary Table 1.

### Theme 1—The Unpredictable and Unprepared Uncovering of Fatigue

For some, fatigue was noticed when the stroke occurred. However, fatigue was often uncovered once home from hospital when returning to usual daily activities, particularly for younger stroke survivors. Fatigue could be physical or mental, or a combination of both. Fatigue could be unpredictable leaving stroke survivors unsure of how much they could achieve a given task, “*You don't know when it's going to hit”* (John^*^). Carers and stroke survivors reported lacking insight into their experience initially, or confused fatigue with other stroke impairments like low mood or emotional lability. This links strongly with the universal sense of feeling unprepared for fatigue.

*I wasn't immediately… showing signs that I was affected by fatigue… At the hospital I got a lot of rest… But then I came home and* (it) *came to the forefront a bit more*. (Kapil)

### Theme 2—Experience and Adjustment to Fatigue Are Personal

Post-stroke fatigue was different to pre-stroke fatigue, but was influenced strongly by pre-morbid personal factors such as self-perceived work ethic and level of activity pre-stroke. Participants acknowledged trying to ignore fatigue, thinking it would ease as recovery proceeded. When it persisted, admitting fatigue was seen as admitting defeat, connected to pre-morbid personal traits such as identifying as a strong and independent person who should not be troubled by a circumstance like fatigue. Stroke survivors reported experiencing feelings of anxiety and fearing the unknown of fatigue, worrying about anticipated events. This manifested in concern about injury if they pushed too hard and worry about exacerbating fatigue in the short and long-term. If improvement was noted, fear of fatigue setback was described.

There was angst about where one could rest when participating outside the home, contributing to restriction in meaningful activities and life roles. Fatigue limited social participation with friends and family, leisure, holidays, accessing the community, vocational work, and familial roles. Frustration was common, heavily influenced by a sense of not meeting the expectations one has of oneself.

*...your inner self, what you expect of yourself and what doesn't happen, it sort of conflicts…….ying and yang on both sides and they're sort of arguing and I'm the one stuck in the middle*. (Lachlan)

Lowered motivation, whilst acknowledged as present in the experience, did not limit initiation of meaningful activities. Stroke survivors overwhelmingly spoke of always trying hard, determined to participate even if that meant spending time resting in days proceeding. As such, there was a notion of fighting with fatigue; participants used language such as “battle”, “trauma” and fatigue having “taken its toll”.

### Theme 3—Being Responsible for Self-Managing Fatigue

Participants described attempting to accommodate and reduce fatigue through self-management. This commenced with a level of acceptance as a starting point for coping and finding some normality and enjoyment in life. Faith and spiritual beliefs helped some cope, when other strategies hadn't worked, more commonly described by stroke survivors born overseas.

*Nothing will help my tiredness except my faith…you always need to have hope…I think my faith keep me alive, keep going*. (Danny)

Self-management strategies included adapting activities and plans to suit fatigue levels each day, pacing and grading activities. Exercise, reducing simultaneous mental stimulants, reducing cognitive and emotional stressors, and minimizing alcohol intake were also helpful. Sleeping helped for some, often utilized mid-morning after usual morning activities. For others sleep made no difference and they still awoke tired.

Universally there was benefit of having predictable, daily routines with rests, such as finding a quiet area to sit or lie down. This involved gathering thoughts, sleeping or just quietening the mind whether physically or mentally fatigued.

…* you gotta rest your brain rather than just shutting your eyes and having a listen to the radio. Yeah, I started turning that off and really trying to switch off for 45 to an hour and I found that really helped*. (Ivan)

With successful self-management, participants measured progress by noting a reduction in fatigue severity, or requiring shorter and less frequent rest. Stroke survivors recounted having no choice but to manage their fatigue because of the negative manifestations. They had to proactively pay attention, be aware of limitations, listen to their body, look after themselves, be flexible and continuously adjust. Carers also highlighted that they felt responsible for supporting self-management of post-stroke fatigue.

*For example, if we're coming here* (rehabilitation*)…then we'll go home I'll prompt him go in your room, just have a lie there. So at night when the kids come home, he's got enough energy to, you know, get up and he'll wash the dishes*. (Georgia—Carer)

### Theme 4—The Complex Juggle of Outpatient Stroke Rehabilitation With Fatigue

Participants noted rehabilitation was challenging when experiencing fatigue. Rehabilitation was described as exhausting at times, resulting in the remainder of the day spent sleeping or resting.

*I concentrate I give it everything but ah yeah after an hour and a, ah pool or something…I ahh, go to sleep for 3 to 4 hours after that*. (Jericho)

Stroke survivors and carers developed strategies to manage this and reported benefits of rehabilitation on fatigue over time, with carers quietly noting the improvement. They tried to plan therapy schedules to optimize fatigue management, requiring rehabilitation services to be flexible to their varied needs. Carers often viewed schedule planning as their duty.

*The more she's doing the exercise…she gets up and wanting to do things. Not get home now and wanna sit down for the rest of the day (like before)*. (Stan—Carer)

*I'd pick the mornings so I know that I can get in there and he's well alert by 12 o'clock, 1 o'clock, we have lunch and he's off to bed. And if they want the afternoon, it will have to be…I've had to make sure that he's well rested before we go*. (Tam—Carer)

Stroke survivors recognized benefits of home sessions and independent exercises for fatigue, but also valued coming to the center for building motivation through personal attention of the therapist and the equipment available. Contrary to all-day exhaustion, many stroke survivors described a second wind of energy after rehabilitation or exercise sessions. This might relate to the physiological effects of exercise, or to a sense of achievement, even if that meant pushing beyond their known limits.

*He started the gym, or when he goes swimming, he has extra energy… he gets tired straight away afterward…but he has like a second wind when he does exercise*. (Tam—Carer)

Fatigue was also felt to negatively impact rehabilitation, resulting in missing part or whole sessions and home practice. Whilst this concerned participants, it was not harbored on, and stroke survivors focused on moving forward through their rehabilitation toward set goals. Goals for many stroke survivors were often related to participation in family-focused activities, particularly for participants from culturally diverse backgrounds, and family members were an additional source of motivation in these experiences.

*The tiredness and the stroke affected my lifestyle. You have to accept it…Because I want to enjoy myself, I want ah to have a conversation with my family, I love my family, they care for me, and they come and see me*. (Bernard)

### Theme 5—Learning About Fatigue Is a Self-Directed Problem-Solving Experience

Stroke survivors reported largely learning to deal with fatigue independent of healthcare professionals. They accessed previous knowledge about their bodies and experiences, and used trial and error to find something that helped, recognizing mental and physical triggers over repeated exposure. Stroke survivors learnt the benefit of preventing exacerbation of fatigue from negative experiences of “overdoing it”. They sometimes learnt from family, friends, other stroke survivors, and internet sources. Some expressed caution when accessing information from the internet, however participants from culturally diverse backgrounds identified the importance of resources in appropriate languages which often came from the internet.

*Like ah even I look in the internet to see what's the best thing to do, get on to the You Tube and check it out, in Turkish*. (Robert—Carer)

Learning from health professionals was dependent on circumstance; receiving education about fatigue was an exception rather than a rule. If the healthcare professional was more experienced, stroke survivors and carers perceived fatigue information was obtained more easily and considered more trustworthy; advice was accepted, and demonstration of how to pace in occupational activities occurred.

Participants felt fatigue was often missed by health professionals. They either “hadn't focused on it”, had not brought it up or put fatigue down to something else such as medication, which the stroke survivor or carer disagreed with. Fatigue was noted by stroke survivors and carers to be missing in stroke education groups. When engaging in outpatient rehabilitation, participants placed less emphasis on learning about fatigue from medical staff they saw only periodically.

*Well they (physio) were mainly concentrating on my physical side…so really haven't focused anything on...fatigue side of it*. (Lachlan)

### Theme 6—Family and Carers Can Support or Constrain Managing Fatigue

Carers appeared to facilitate the uncovering of fatigue and the journey to self-management, reported by both stroke survivors and carers. Carers identified they could enable learning through reinforcing education when received, researching independently, or noticing fatigue triggers. Rather than performing a task for the stroke survivor, carers assisted with task completion or monitored pacing, allowing stroke survivors to gradually increase capacity.

Carers adopted rehabilitation scheduling and routine planning responsibilities, played a protective role, and often took a large physical load. Carers often reported frustration at setting pacing boundaries for fatigue management that stroke survivors did not adhere to. Many stroke survivors recognized the role of carers in fatigue management and worried about those without carers. Several carers expressed anxiety about how they would continue this role once formal rehabilitation finished.

*I wanted to get her back into life again. So we've always shared the workload and we'll remain to share it. At the moment with her fatigue and that, I'll do a bit more, for now. But she'll get back into it I can see. I just gotta teach her to manage*. (Stan—Carer)

*I'm the gatekeeper*. (Maria—Carer)

Some stroke survivors reported family members could be dismissive of fatigue and possess unrealistic expectations of them, contributing to further distress. This was predominantly reported by stoke survivors with minimal stroke deficits or by young stroke survivors within a busy family unit. Universally, the invisibility of fatigue was perceived as influencing the expectations of others, “I suppose it's like the silent killer.” (Kapil)

Carers also identified they can alter their existing approach to be more positive in supporting the stroke survivor with managing fatigue.

*I remember* (physiotherapist) *said something like ‘Oh sometimes family is hard because sometimes family doesn't believe that you're tired'. I'm thinking oh my god that's me…I get frustrated…he's like ‘Oh I'm tired' and it's like ughh. So ever since I heard that I was a bit more mindful I gotta change. Because before I was pushing him*. (Georgia—Carer)

## Discussion

In this qualitative study we described the experience of post-stroke fatigue during outpatient rehabilitation from the perspective of stroke survivors and carers. Utilizing a descriptive, phenomenological methodology, six themes were identified: The unpredictable and unprepared uncovering of fatigue, Experience and adjustment are personal, Being responsible for self-management, The complex juggle of stroke rehabilitation with fatigue, Learning about fatigue is a self-directed problem-solving experience, Family and carers can support or constrain managing fatigue.

This study provides empirical data to support expert-consensus recommendations in international stroke guidelines (34–36). It provides new insights into the powerful positive and negative influence of carers on the fatigue experience for stroke survivors. It also reveals that stroke survivors largely learn to manage fatigue independent of their healthcare team in outpatient rehabilitation. Some of our findings are consistent with prior studies that similarly found fatigue was complicated by its invisibility, and that stroke survivor and carers' lack of knowledge about fatigue contributed to difficulty coping, adding emotional distress (12). Individual qualitative studies have also reported stroke survivors feel restricted, frustrated (11), unprepared, and that fatigue is most noticeable soon after hospital discharge (37, 38). The perceived benefits of pacing and physical activity have previously been described (10, 12, 14, 37–40).

Our study sought to specifically investigate the fatigue experience during outpatient stroke rehabilitation, providing novel insights. It was not always clear in other studies whether people were participating in rehabilition (12). In two recent studies investigating stroke and/or brain injury fatigue experience only 24–35% of participants were receiving outpatient rehabilitation (38, 41). In our study stroke survivors used a range of strategies to optimize energy levels and adapt their rehabilitation schedule to their fatigue. However, negative impact of fatigue on rehabilitation participation and progress was acknowledged by participants. Current evidence supports high-dose, repetitive exercise to optimize rehabilitation outcomes post-stroke (7, 8). However, early phase fatigue is a predictor of physical health 18 months after stroke (42), suggesting fatigue may interfere with long-term recovery. Recent experiential evidence indicates fatigue can impair motor-skill learning (43) and slow movement reaction time (44). In healthy individuals, a finger-tapping task performed to fatigue resulted in deleterious motor skill performance for up to 2 days (43). Further research is required to understand this impact, and how high-dose rehabilitation can be optimized for those with fatigue. In the meantime, healthcare professionals should be aware of possible implications of fatigue on neural plasticity-driven motor-skill attainment. At a minimum, as emphasized by participants in this study, healthcare professionals should be scheduling therapy at optimal times for the individualized needs of each stroke survivor. In our experience this is rarely prioritized in practice. Instead stroke survivors fit into the confines of busy outpatient rehabilitation diaries set between rigid meal breaks and start and finish times. Alternative service models could be considered, especially given telehealth has been well established in most countries since the COVID-19 pandemic. Well-coordinated blended models incorporating telehealth, community outings, home-based and center-based multi-disciplinary care, giving stroke survivors and carers choice may be efficacious and are advocated by the illustrative quotations from this study (see Supplementary Table 1).

We found fatigue may not be identified, and education not provided by health professionals most qualified to do so. Consistent with previous studies, stroke survivors largely learnt to manage fatigue themselves, using trial and error (37). Interestingly, healthcare professionals rate post-stroke fatigue research implementation as a lower priority compared with consumers (45). If health professionals do provide knowledge about post-stroke fatigue they conceptualize it in divergent ways (46). This highlights a need to educate health professionals about post-stroke fatigue (34, 46) to ensure rehabilitation services provide a consistent, structured, supportive and motivational environment for fatigue management. Recognition of fatigue through periodic screening is important (34), especially given lack of acknowledgment of fatigue by health professionals and family is a further burden for stroke survivors (12). As highlighted by participants in this study, fatigue may not be present at stroke onset, or may be missed by healthcare professionals involved along the continuum of care, suggesting subsequent screening and questioniong in the weeks and months post stroke is important. Finally, attention should be paid to providing language and culturally appropriate educational resources from trustworthy sources.

We have shown that carers can faciliate further learning and self-management of post-stroke fatigue, supporting generation of a routine including rests, rehabilitation scheduling and paced home practice. In addition, family can provide motivation, particularly for people of cultural backgrounds where emphasis is placed on collective family life (47). Healthcare professionals could maximize carer-facilitated fatigue management by recognizing family-centered goals as well as positive family engagement in rehabilitation and care. For example, stroke survivor social participation with their family may be an opportunity for carers to facilitate pacing. In contrast, when carers lack understanding about fatigue, or no carer is available, stroke survivors reported distress and frustration. This was particularly evident for participants of working age who had dependent children and working partners. Social support and marital status have previously shown mixed associations with post-stroke fatigue (48). Our findings highlight carers play an important role and must be considered in the clinical management and future research of post-stroke fatigue. Where stroke survivors have no carer, strategies to replace this support should be considered such as formal paid carers to assist pacing and reinforcing education and self management.

This study utilized a robust descriptive phenomenological methodology, with evidence of a clear decision trail, bracketing and reflexive practices. A key strength was the inclusion of stroke survivors with aphasia and a culturally diverse group of stroke survivors which is reflective of stroke survivors at the study site.

### Limitations

Results of this study are potentially not representative of stroke survivors who were not receiving intensive outpatient physiotherapy services, and therefore limits understanding of the experience for those without physical deficits. However, other studies with community-dwelling stroke survivors reported similar experiences of searching for answers due to a lack of information about fatigue from health professionals (37, 40). We have extended understanding by describing where and how stroke survivors and carers sought information. Interviewing stroke survivors and carers together may have restricted participant comfort to speak freely. We set out to understand stroke survivor experiences and the compelling insights about caring for a stroke survivor with fatigue highlight carer burden should be explored in future research. The Fatigue Assessment Scale is one of several existing fatigue measures. Limitations in accurate detection and measurement of available fatigue measures have been highlighted in the literature (49) so we may have unintentionally omitted other stroke survivors with fatigue. Sample size was small but was justified by our in-depth analysis of strong, clear interview dialogues, and a sample that had dense specificity of information relevant for the study aims, providing adequate information power (32). Qualitative research results are not designed to be reproducible as eliciting participant lived-experience in constructivist-based descriptive phenomenology is situation-specific (50), reflecting the voices of recruited participants. We also cannot make inferences about the typical experience of specific cultures as our participants were from a broad range of cultural groups.

## Conclusion

This study has provided insights for clinical practice. Stroke survivors largely learnt to manage fatigue independent of their healthcare team in outpatient rehabilitation, and carers can powerfully influence the experience both positively and negatively. Healthcare professionals should consistently screen for fatigue and provide stroke survivors and carers with individualized, language and culturally-appropriate support and education regarding fatigue impact, and practical strategies to manage. Healthcare professionals can maximize carer-mediated fatigue support where family are involved in rehabilitation care, or play an important role in patient participatory goals. Rehabilitation services should provide flexible models of care and session scheduling to suit the individualized needs of the stroke survivor with fatigue. Further research is required to develop management interventions and optimal rehabilitation programs for stroke survivors with fatigue, and explore whether the experience is different for specific cultural groups.

^*^ Names used throughout this manuscript are pseudonyms.

## Data Availability Statement

The raw data supporting the conclusions of this article will be made available by the authors, without undue reservation.

## Ethics Statement

The studies involving human participants were reviewed and approved by Western Health Low Risk Ethics Panel HREC/18/WH/47137 and Australian Catholic University Ethics Committee 2019-17R. The patients/participants provided their written informed consent to participate in this study.

## Author Contributions

EB designed the study, conducted and transcribed all the interviews, was responsible for data analysis, and manuscript preparation. SK supported study design, conducted data analysis, and contributed to writing and editing the manuscript. CS and KH reviewed the study design, audited the research process, verified data analysis, and contributed to manuscript preparation. All authors contributed meaningfully to the preparation of this manuscript, contributed to the article, and approved the submitted version.

## Funding

This work was supported by a Western Health Research Grant.

## Conflict of Interest

The authors declare that the research was conducted in the absence of any commercial or financial relationships that could be construed as a potential conflict of interest. The reviewer DS declared a past co-authorship with one of the authors SK to the handling editor.

## Publisher's Note

All claims expressed in this article are solely those of the authors and do not necessarily represent those of their affiliated organizations, or those of the publisher, the editors and the reviewers. Any product that may be evaluated in this article, or claim that may be made by its manufacturer, is not guaranteed or endorsed by the publisher.
