# Supplementary material for: “I Give It Everything for an Hour Then I Sleep for Four.” The Experience of Post-stroke Fatigue During Outpatient Rehabilitation Including the Perspectives of Carers: A Qualitative Study
Source: Front Neurol. 2022 Jun 2;13:900198. doi: 10.3389/fneur.2022.900198 (PMC9201517; doi:10.3389/fneur.2022.900198)
Supplement: Supplementary file 1 [file Table_1.pdf]

Supplementary Material

Table 1. Additional illustrative excerpts

Theme 1 – The unpredictable and unprepared uncovering of fatigue	
Fatigue uncovered once home from hospital	*"Perhaps not in the hospitals…when I first got home then I was here* (rehab center), *everything was go go go.. So I probably right at the start didn't feel it straight away."* Neva
Confusion with other symptoms	*"I suppose that will come out in other ways too. I might be ah slightly more argumentative, or I don't want to play* (with daughter)." Kapil
Physical and mental or a combination	*"I'll split it into physical and mental fatigue…um no I think they relate to each other."* Francesco<br>*"If I'm tired physically my brain is also more tired. I think it's all connected."* Danny
Unprepared	*"I didn't expect it. When it hit me it was friggin' what's this shit? What's wrong with me? You just don't know what to prepare yourself for."* John<br>*"I didn't think it was going to be a major problem ah the way it is, yeah."* Jericho
Theme 2 – Experience and adjustment are personal	
Different to pre-stroke fatigue and influenced by premorbid personal factors	*"It's definitely different. I think um, the way my fatigue affects me is ah, is fairly, I suppose heightened. Beforehand it didn't affect me too much. It was something that I didn't really consider. I know my body or how I was I could push through it really easily."* Kapil<br>*"I'm a doing person you know…I'm a do-er."* Olga
Not wanting to admit fatigue	*"I felt like I was admitting defeat if I admitted it."* Lachlan
Anxiety about unknowns of fatigue	*"But yeah, it makes me worry, I go oh jeez am I getting good and then in 6 months I'm going to have a turn you know, the wheels are going to turn the other way yeah am I doing too much?"* Ivan<br>*"I think he's a little bit fearful of the fatigue and being tired."* Susan - Carer
Loss of participation and loss of role	*"So the grandkids come around, and they wanna go out the backyard and play and she* (wife) *says 'Go out and watch the kids'…but you're just, you're just sitting there watching them, I can't chase them, it would just exhaust me too much."* John<br>*"I loved doing gardening but I can't do it because I get tired. You know, sitting down and talking for a period of time I get tired as well. I've learnt now when there's a few people here and I'm tired…I got to bed, I just leave everyone and go to bed."* Bernard<br>*"Once I got to bed, I don't want to get up again, so ah I have to eat me tea in bed and things like that. Whereas I'd like to sit up and eat it at the table."* Jericho
Frustration and not meeting self-expectations	*"I just sit on the couch and I hate it. Because I haven't got the energy to get up and do what I want to do."* Rose<br>*"When I've physically fatigued the challenge is then to respond to it, to answer it, in my own head. That was frustrating."* Francesco
Fighting with fatigue, lowered motivation but not impacting initiation of activities	*"I just kept walking, didn't sort of worry when I was getting tired, but just kept pushing you know…There hasn't been a day I haven't been out of bed, you know, um..and have a shower and go for a walk. I've never stayed in bed all day long because of you know... I've gotta get up and do what I gotta do"* John<br>*"I have to push myself to do what I have to do until I come home and have a rest through the night and the next day I sleep in a bit, an extra hour or two.*" Bernard
Pushing through to participate in meaningful activities	*"That was what I set myself, one dance for him for his birthday. Um but it was wonderful, all the hall came and said hello to me. The buzz must have gone round…It was the best thing I could have done…*(I was) *stuffed but that's alright I urn it, earnt it and enjoyed it."* Olga

Theme 3 – Being responsible for self-managing fatigue	
Acceptance for coping, faith	*"It's just something that I've gotta learn to cope with these days as you know, part of the illness that I got hit with."* Lachlan<br>*"Nothing will help my tiredness except my faith…you always need to have hope. I think my faith keep me alive, keep going."* Danny
Strategies for self-management	*"The extra exercise that I'm doing is definitely helping."* Kapil<br>*"The (psychologist) helped me a lot yeah. I just do a lot of breathing you know. Try to block all my negative stuff, my thinking...I listen to music."* Ahmed<br>*"Try not to do too many things in one day. Space them out over a period of time…pace myself."* Francesco
Routine with rests, often carer-led	*"For example, if we're coming here* (rehabilitation)*…then we'll go home I'll prompt him go in your room, just have a lie there. So at night when the kids come home, he's got enough energy to, you know, get up and he'll wash the dishes. So just breaking things. Having rests in between."* Georgia - Carer<br>*"Say sweeping the floor… doing this for 10 minutes or something but I find I've gotta sit down and rest for a while and, before I take it up again."* Neva<br>*"You are resting little bit, the brain not think…when I wake up, little bit fresh."* George
Proactive, aware of limitations, adjustment of expectations & plans	*"It makes you think, makes you evaluate yeah, what are you doing, yeah don't do too much, don't overdo it…you've gotta manage it quite diligently."* Ivan<br>*"That's only because I'm aware of what I'm doing and where I am at the time so I'll adjust. I'll try not to get too tired…you know your own body gives you signals."* Lachlan
Theme 4 – The complex juggle of outpatient stroke rehabilitation with fatigue	
Rehab is motivating and can be energizing as well as exhausting	*"I go on the bike, it hurts me, but, at least when I get home, it helped me a bit to do more at the house you know so."* Rose<br>*"I just find them really interesting* (therapy sessions). *They'd (rehab professionals) really encourage me, push me along but then it sounds like I get fatigued for nothing isn't it? I don't know. I just feel that when I'm doing things at home it must be, that I'm really, perhaps it's because I haven't got the encouragement."* Neva
Planning therapy within constraints of fatigue and needing the rehab service to be flexible, often carer-driven	*"If he had speech at 9, OT at 10, Physio at 11, it's too much for anyone that's had a stroke and suffering fatigue."* Katherine - Carer<br>*"In conjunction with the physios and OT you know, do something Monday, have Tuesday off, have something Wednesday, have Thursday off…What I found I was asking them to come home, to try to come to the home, which they were ok with you know and I'd come in to do hydro and a physio session on Tuesday…*(because) *I'd do more here."* Ivan<br>*"He's tired yeah. He's too many things one day. Can't remember what you said (neuropsychologist), what you said (OT) you know? Getting it all mix up. Three groups one day is too much."* Sharon - Carer<br>*"He's always been a morning person, an early riser. If he has…afternoon sessions I've noticed that those sessions aren't as successful."* Susan - Carer
Negative impact on rehab – missing sessions, restrictions to parts of therapy	*"At times I find it really trying to actually show up for my rehab…I really hate missing any classes. If it doesn't work out that's because I really am too tired. I feel that it's inhibiting my progress."* Lachlan<br>*"I never missed appointment…Because I'm very keen to get better and improve. To some extent I think* (fatigue) *has* (impacted progress)*, but I try to overlook that and get on with it and do what I have to do."* Bernard
Focus on goals, often related to participation in family-focused activities, especially in culturally-diverse participants	*"The tiredness and the stroke affected my lifestyle. You have to accept it…Because I want to enjoy myself, I want ah to have a conversation with my family, I love my family, they care for me, and they come and see me."* Bernard<br>*"I'll set myself a target…that target will drive me mentally and physically. I'm gonna manage fatigue."* Francesco<br>*"I loved sewing…my daughter's dress I do up. Because she was going to wedding, she needs to, I don't want her spend too much money. I can do it. Yeah. I push myself."* Maida
Measuring improvement, self and carer-noted	*"I'm finding working wise I'm not taking as many breaks anymore."* Ivan<br>*"She used to sit down after she'd done something…totally shattered. Not now. She'll sit down and say, 'I'm tired', I'll just have a cup of coffee. Then she'll get back up again, right. So her energy is generated a lot more quickly."* Stan - Carer
Theme 5 – Learning about fatigue is a self-directed problem-solving experience	

Learning from knowledge about own body, previous experience, family & friends	*"(Another stroke survivor) said you're the only one I know that's got this, come and talk to me…we'd compare how we were going and it was so nice for me to talk to him."* Neva<br>*"I'm learning from my body."* Francesco<br>*"No uh at work ah, truck drivers and things like that, I used to do a course of fatigue management at ah VicRoads ah I went a few courses."* Jericho
Trial and error	*"Like I say it's trial and error. So you know, if this works, cool, and if it doesn't, go on to plan B. Just keep trying…I've got 26 letters to go through so."* Lachlan<br>*"It's a question of trying something to see if it's gonna work and if I, when I try once and twice and saw that it made a difference to my routine I said that's it, that's the way I should go about it."* Bernard
Learning to prevent exacerbation	*"If I try to push through it, I know that I can do it, but I will definitely see the side effects from my fatigue later that day, or maybe the next day…If I try to postpone it, it will definitely come back to bite me harder."* Kapil<br>*"He would push himself, push push push push, then he would just explode and I'm like you're too tired go in your room."* Georgia - Carer
Internet sources	*"I was reading on the stroke foundation website…I was reading the stroke foundation Facebook page."* Ivan<br>*"Well being the type of person I am, I mean my curiosity gets the better of me and if I can't find ways of getting help, I'll Google it…I don't want to become a hypochondriac so I sort of have a look and then I'll get away from it."* Lachlan<br>*"I learn from the books as well. Like ah even I look in the internet to see what's the best thing to do, get on to the You Tube and check it out, in Turkish."* Robert - Carer
Learning from health professionals, level of experience matters	*"…the advice of you know (occupational therapist) and (occupational therapy assistant)…what to do and what to notice...you know for example doing the dishes, 'Right do the dishes, put the timer on' yep. You know 'How long's it take you to do the dishes, are you feeling fatigued after you do it?' 'Yeah I am.' 'Let's…turn the timer off, go and sit down. How long was that? Right it was 10mins. Ok well let's try to build that up to 15 next week.'"* Ivan<br>*"The ones who had been working in rehab for quite a while, they would recognize the physical signs better, the tiredness. They could see it on his face."* Susan - Carer
Fatigue was missed by health professionals, putting fatigue down to something else, missing from education	*"No I don't think they got much information. Even those sessions* (stroke education group) *we did, you didn't get really a lot of information. It was good for other things, cause I learnt a lot…but it didn't cover a lot on fatigue."* Katherine - Carer<br>*"I think a lot put it down to since the seizure she's been on Keppra…But she's had it all the time since the stroke before the Keppra. So to me it's not the Keppra."* Stan - Carer<br>*"We're taught how to manage cognition, speech, physical issues but aren't taught how to manage fatigue."* Georgia - Carer
Theme 6 - Family and carers can support or constrain managing fatigue	
Carers facilitate uncovering, journey to self-management, enabling learning, implementing proactive management plans	*"It's not her chores or my chores we do it together and um so that's helpful because I'd get her up and then she goes, 'I feel tired now' after 5 minutes. 'Go sit down, I'll make you a cuppa' and I'd continue with this job. Cooking, the same so we gradually build on that and the fatigue ah has come lower."* Stan - Carer<br>*"I have to make a conscious effort that um to just space out his um activities. And if I notice I'll just tell him go and rest even though you're not having a nap… Cause otherwise it will just, the whole day and the next day he'll just be tired…For example, tomorrow, we've got a dinner tomorrow night so in my head I'm already thinking… if he does too much during the day, at night he'll just be kinda just zone out or be too tired to interact with the other people."* Georgia - Carer<br>*"(My wife) me motivate, doing them make me laugh, make me energy."* Danny
Carer frustration - stroke survivor not adhering to pacing boundaries	"*I told her off yesterday right. Because she started getting back to what she was. Because she felt better once she left here* (rehabilitation center)*. She went and done that, she went and vacuumed, she hung the washing out, she washed the dishes and wouldn't stop, then I saw it in her face, she was shattered*." Stan - Carer
Worrying about stroke survivors without carers	*"At the end of the day if you don't have someone to support you and give you the support you need like my (carer), it makes a lot of difference. If you don't have anyone who cares about you, you've got no chance to get better."* Bernard
Dismissive, unrealistic expectations, distress	*"You know my wife…in her mind I think she thought right (stroke survivor) is back, right we're back now we're right, but yeah it was yeah, it was far from it."* Ivan<br>*"Because of my fatigue I...don't pay enough...attention to my wife, and the attention to my daughter. I think all our expectations are pretty high and because I'm not meeting them, it feels like I'm a bit of a failure. If I don't remind them every now and again they will just expect me to…deal with things like I used to."* Kapil
Invisibility	*"See we've got a problem with people we know look at (stroke survivor) and think there's nothing wrong with him...that's how our friends are perceiving him, there's nothing wrong with him, because he's not the typical stroke person."* Katherine - Carer<br>*"Nobody understands because I look good. Nobody understand I can't do it anyone, I can't standing."* Maida
